# SPIONs for cell labelling and tracking using MRI: magnetite or maghemite?†
†Electronic supplementary information (ESI) available: pXRD, DLS, zeta potential, TEM, TGA, MRI and XANES. See DOI: 10.1039/c7bm00515f


**DOI:** 10.1039/c7bm00515f

**Published:** 2017-11-23

**Authors:** Michael Barrow, Arthur Taylor, Ana M. Fuentes-Caparrós, Jack Sharkey, Luke M. Daniels, Pranab Mandal, B. Kevin Park, Patricia Murray, Matthew J. Rosseinsky, Dave J. Adams

**Affiliations:** a Department of Chemistry , University of Liverpool , Liverpool , UK . Email: Dave.Adams@glasgow.ac.uk ; Email: rossein@liverpool.ac.uk; b Centre for Preclinical Imaging , Institute of Translational Medicine , University of Liverpool , Liverpool , UK; c MRC Centre for Drug Safety Science , Department of Clinical and Molecular Pharmacology , University of Liverpool , Liverpool , UK; d School of Chemistry , College of Science and Engineering , University of Glasgow , Glasgow , G12 8QQ , UK

## Abstract

Although there is extensive literature covering the biomedical applications of superparamagnetic iron oxide nanoparticles (SPIONs), the phase of the iron oxide core used is not often taken into account when cell labelling and tracking studies for regenerative medicine are considered. Here, we use a co-precipitation reaction to synthesise particles of both magnetite- (Fe_3_O_4_) and maghemite- (γ-Fe_2_O_3_) based cores and consider whether the extra synthesis step to make maghemite based particles is advantageous for cell tracking.

## Abstract 2

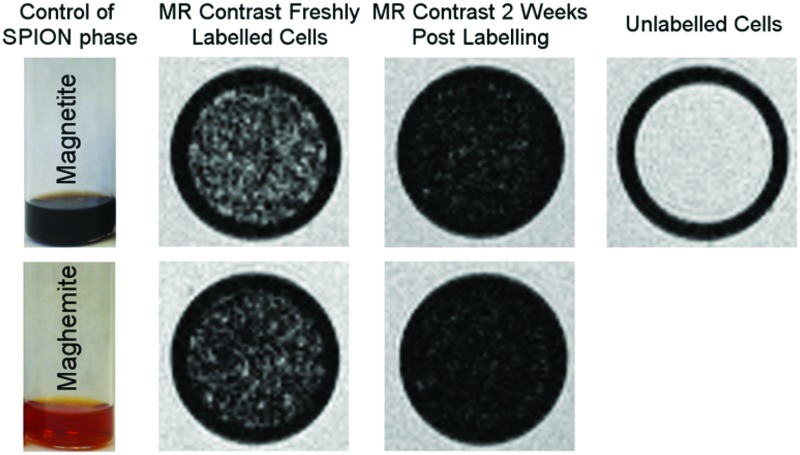
SPIONs consisting predominantly of magnetite or maghemite display distinct chemical stability in solution but equivalent imaging properties when in cells.

## 

Superparamagnetic iron oxide nanoparticles (SPIONs) are commonly used as cell tracking agents using magnetic resonance imaging (MRI).^1^–^3^ SPIONs enhance contrast by altering the transverse relaxation time of protons contained within surrounding tissue. Many studies involving iron oxide nanoparticles for cell labelling applications fail to provide clear characterisation of the core itself due to the well-known difficulty in quantifying the ratio of magnetite and maghemite.^4^–^6^ Both phases involve the occupation of octahedral and tetrahedral sites within a close-packed array of oxide anions.

Magnetite Fe_3_O_4_ is a spin polarised mixed valence metal where 2/3 of the iron sites are Fe^3+^ and 1/3 Fe^2+^. Fe^2+^–Fe^3+^ intervalence charge transfer causes absorption throughout the UV-vis and IR regions and hence magnetite appears black in colour.^7^ Magnetite can slowly oxidise in aqueous conditions or oxygen-containing environments at high temperatures to the all-Fe^3+^ maghemite. However, oxidisation proceeds a lot slower when stored as a powder at room temperature.^8^,^9^ Maghemite (γ-Fe_2_O_3_), which is an oxidation product of magnetite (Fe_3_O_4_) at temperatures below 200 °C, shows very little solution absorbance above 700 nm and appears brown-orange in colour.^9^ Both phases contain an inverse spinel face-centred cubic oxide lattice with almost identical unit cell dimensions with lattice parameters of *a* = 8.39 Å for Fe_3_O_4_ 10 and either *a* = 8.34 Å or 8.35 Å for γ-Fe_2_O_3_ depending on method of synthesis,^11^,^12^ making it very difficult to distinguish using powder X-ray diffraction (pXRD) alone.^7^,^13^ Maghemite is often termed Fe(ii) deficient or fully oxidised magnetite.^13^ Both magnetite and maghemite are ferrimagnetic at sizes >20 nm.^1^ However, at sizes below 20 nm, they both exhibit superparamagnetism where they are only magnetised under the influence of an external magnetic field, with zero coercivity, meaning a reversal of field is not required to reduce magnetisation to zero.^14^ This property, as well as their relatively low toxicity, makes these <20 nm SPIONs useful as contrast agent for MRI.^1^ For regenerative medicine therapies, SPIONs have been extensively used pre-clinically as cell tracking agents^15^,^16^ and have found application in clinical research.^1^ The rationale behind these studies is the monitoring of the localisation of SPION-labelled cells (*e.g.*, stem cells or macrophages) upon injection into the host to better understand their biodistribution and therapeutic mode of action. Importantly, this imaging technique is not hindered by penetration depth or spatial resolution limitations and can give detailed anatomical information.^15^,^17^,^18^ The SPIONs consist of an iron oxide core surrounded by a polymer shell that controls the core size and determines the colloidal stability and cellular uptake of the SPIONs.

For most studies involving iron oxide nanoparticles as cell labelling agents, characterisation of the iron oxide core *i.e.* the amount of magnetite *vs.* maghemite is not often quoted and the nanoparticles are often cited as being Fe_3_O_4_ or γ-Fe_2_O_3_ SPIONs without sufficient evidence to support either phase being dominant. In general, so-called Fe_3_O_4_ cores are more widely studied as contrast agents for cell tracking due to their easier synthesis. Even though Fe_3_O_4_ has a slightly larger saturation magnetisation than γ-Fe_2_O_3_, this is not likely to have a major effect on the MRI contrast of exogenously labelled cells, as previous studies showed that particles with different saturation magnetisation all have very similar relaxivities upon cell internalisation.^19^ For increased contrast, it is necessary to increase uptake, and for stem cells this is usually achieved by use of a cationic polymer, which also renders SPIONs stable in water and cell culture medium.^19^

To access pure phase γ-Fe_2_O_3_ nanoparticles, Fe_3_O_4_-containing materials are usually synthesised first followed by an oxidation step. It has been claimed that the breakdown of Fe_3_O_4_ to γ-Fe_2_O_3_ could lead to toxicity issues in stem cells, where Fe^2+^ ions released in the endosomes or lysosomes could catalyse Fenton-type reactions leading to the formation of toxic radicals.^20^ Some researchers prefer to oxidise particles before cell labelling, which would avoid the problem of *in vivo* radical formation, and have suggested that γ-Fe_2_O_3_ nanoparticles are more stable to degradation in the acidic medium present in a lysosomal environment.^21^ Therefore, there is a need to directly relate the synthesis conditions of SPIONs with their core composition and overall efficiency as cell tracking agents for MRI.

Whilst there have been studies quantifying the amounts of magnetite or maghemite in nanoparticles in general,^4^ as well as magnetite oxidation,^8^,^9^ there are very few examples in the literature linking this structural characterisation with their MR performance in cells. As stated above, because of their very similar unit cell dimensions it is difficult to distinguish between the two phases by pXRD alone due to peak broadening associated with very small core sizes. Some of the other methods used to distinguish between the two phases are UV-vis NIR spectroscopy,^9^ X-ray absorption near edge spectroscopy,^22^ Fourier transform infra-red spectroscopy (FTIR),^4^ Mössbauer spectroscopy^6^ and Raman spectroscopy.^23^ Raman spectroscopy can sometimes be problematic if the laser power is too high as it can actually oxidise magnetite.^23^ Park *et al.* compared magnetite and maghemite nanoparticles for labelling alveolar macrophages and concluded that maghemite is the less toxic of the two.^24^ However, definitive characterising data to establish the identities of the iron oxide phases in the cores of these SPIONs were not presented, colloidal stability was not quoted and the hydrodynamic diameters and zeta potentials were quite different in the two cases, which could affect both uptake and toxicity.

We propose that to accurately compare SPIONs based on different dominant iron oxide core phases for cell labelling and tracking using MRI, we must attempt to control other physicochemical properties which could affect their toxicological profile. Here, we have synthesised SPIONs with different dominant phases of iron oxide in the core and report their hydrodynamic diameter, zeta potential, polymer coating and content and core size.

We used a synthesis method similar to one we have previously employed, where we showed that varying the ratio of fluorescein isothiocyanate (FITC)-diethylaminoethyl (DEAE) dextran to iron salts used in the co-precipitation reaction can control the core size of synthesised SPIONs and their uptake in mouse mesenchymal cells and macrophages.^19^,^25^,^26^ To synthesise SPIONs with a magnetite core, we used a ∼1 : 1 DEAE-dextran (non FITC-containing) polymer to iron salt ratio and carried the reaction out under nitrogen (see ESI† for full Materials and methods). The resulting SPIONs were oxidised by exposing the magnetite samples to air, attaching a reflux condenser and heating at 110 °C in air for a period of 5 hours. During the synthesis, aliquots taken at various time points (Fig. 1) show the characteristic change in colour from black (magnetite) to orange (maghemite) and the corresponding loss in absorbance above 650 nm. Aliquots were taken at time *t* = 0 hours, which is directly after the synthesis under nitrogen, then at *t* = 1 hours, *t* = 3 hours, and *t* = 5 hours after the samples were exposed to air and heated to reflux.

**Fig. 1:**
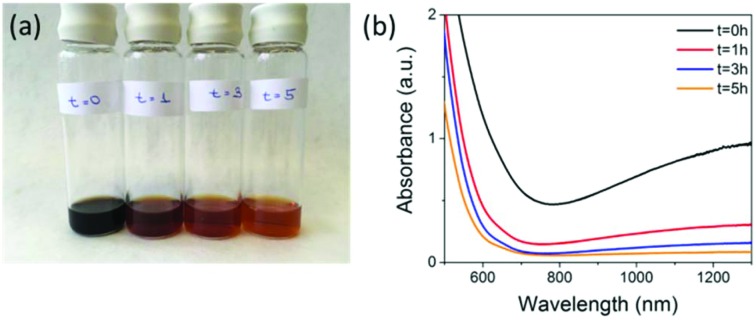
(a) Picture showing 5 mL aliquots taken from the reaction mixture at (from left to right) *t* = 0, 1, 3 and 5 hours post-exposure to air. (b) The corresponding loss of absorbance above ∼650 nm for the aliquots removed from the reaction mixture.

These samples were then stored under nitrogen until further measurements were carried out. The loss in absorbance and colour change with time were similar to what was observed by Tang *et al.* over the same time period, where they claimed full conversion to maghemite.^9^ Samples *t* = 0 hours and *t* = 5 hours were selected to be compared for cell labelling as *t* = 0 hours had the highest NIR absorption, indicating the highest content of magnetite present. However, it is not possible to calculate the absolute amount of magnetite from this measurement alone. Both materials have characteristic iron oxide reflections by pXRD with lattice parameters of *a* = 8.37 Å for *t* = 0 hours and *a* = 8.35 Å for *t* = 5 hours indicating the unit cell has got smaller. The particle core sizes are very similar 8.8 nm for *t* = 0 hours and 9.1 nm for *t* = 5 hours, as calculated using the Scherer equation (Table 1 & Fig. S1†). The hydrodynamic diameters were 86.7 nm for *t* = 0 hours and 64.4 nm for *t* = 5 hours in 0.01 M NaCl (Table 1, Fig. S2 & Table S2†) and the overall polymer coverage, which was calculated using thermogravimetric analysis (TGA) to be 82.1% for the sample *t* = 0 hours and 80.2% for the sample *t* = 5 hours (Table 1 and Fig. S3†). The slightly lower polymer content for the 5 hours sample could be a result of the extra oxidation step causing some polymer to detach from the surface, which also resulted in a decrease in the hydrodynamic diameter from 86.7 nm to 64.4 nm. Importantly, the surface charge (apparent zeta potential) is also maintained with a minimal drop from 19.9 mV for *t* = 0 to 19.0 mV for the *t* = 5 hours sample. Transmission electron microscopy (TEM) showed that particles had very similar size and morphology (Fig. S4†). Magnetisation curves (Fig. S5†) showed a decrease in saturation magnetisation from 115 emu g^–1^ [Fe] to 95 emu g^–1^ [Fe], a further evidence of oxidation as magnetite has a larger saturation magnetisation than maghemite.^7^ We have quoted values in emu g^–1^ [Fe], as this is a more representative quantity when the precise phase present is unknown.^19^

**Table 1:** Properties of SPIONs at *t* = 0 hours and *t* = 5 hours post-exposure to air

Sample	Core size pXRD (nm)	Hydrodynamic *Z*-avg in 0.01 M NaCl (nm)	Zeta potential in 0.01 M NaCl (mV)	Polymer content by TGA (%)
*t* = 0 hours	8.8	86.7	19.9	82.1
*t* = 5 hours	9.1	64.4	19.0	80.2

To investigate the phase of the iron oxide core in more detail, we used the same co-precipitation reaction as before, with the only difference being that no polymer is used in the synthesis. This protocol also yields particles with core sizes of around 10 nm. X-ray absorption near-edge spectroscopy (XANES) was used to determine the average oxidation state of iron in samples prepared at *t* = 0 hours and at *t* = 5 hours with respect to commercially available standard materials with known stoichiometry (Fig. 2a). When the edge energy was plotted with respect to the oxidation states (Fig. 2b) of the standard materials, the sample prepared at *t* = 0 hours had an average iron oxidation state of +2.8, which confirms that the majority phase is magnetite (60%) with 40% being maghemite. For the sample prepared at *t* = 5 hours, the edge energy matches that of γ-Fe_2_O_3_, which gives an average iron oxidation state of +3 confirming that the extra oxidation step converts all the iron oxide to pure maghemite. These edge values are similar to iron oxide values published elsewhere.^22^

**Fig. 2:**
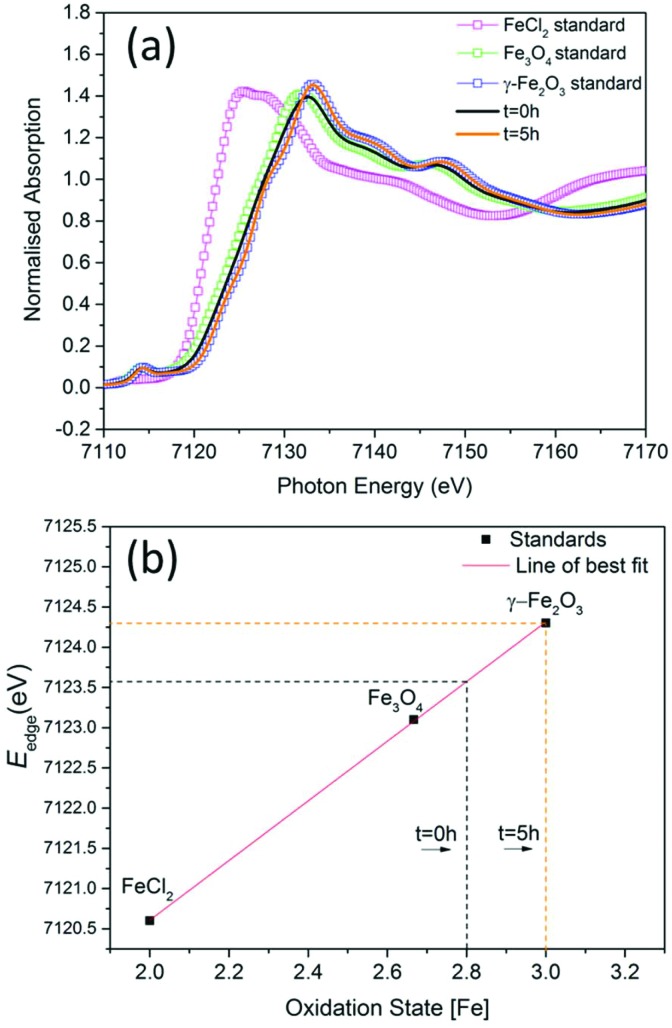
(a) The experimental Fe K-edge XANES spectra of samples prepared at *t* = 0 hours and at *t* = 5 hours and standard materials (smaller energy range shown in ESI Fig. S6†). (b) Edge energies plotted with respect to oxidation states of standards.

When SPIONs are taken up by stem cells, they have been shown in numerous studies to be preferentially localised within endosomes, which have local pH in the 4.5–5.5 range.^27^ However, assessing the stability of iron oxide nanoparticles within cells can be difficult. Some researchers have suggested using acidic buffers containing citrate as a lysosomal mimic for understanding particle degradation.^28^ Whilst this technique is employed for comparative purposes and can give an idea if one sample is ‘more stable’ than another, it must be noted that the method does not reproduce the exact conditions found in lysosomes and thus, it can be difficult to correlate such degradation data with the particle's stability in cells or *in vivo*. In general, the citrate assay is likely to overestimate the kinetics of particle dissolution, where published data suggest nearly full dissolution after just a few days.^27^ On the other hand, when actual cells are imaged post SPION labelling, significant contrast by MR is often observed for weeks.^27^ In Fig. 3a, we show dissolution of the SPIONs prepared at *t* = 0 hours and at *t* = 5 hours in citrate buffer at pH 4.5. The SPIONs prepared at *t* = 0 hours dissolve at a much faster rate than those prepared at *t* = 5 hours. After 48 hours exposure to the citrate buffer solution, for example, the SPIONs prepared at *t* = 0 hours are more than 90% dissolved whereas the samples prepared at *t* = 5 hours are only 30–40% dissolved. In Fig. 3b, we have compared solution relaxivity, which is often quoted as a means of understanding SPION efficiency as MR contrast agent, before and after 48 h exposure to citrate buffer. As we and others have previously shown that 
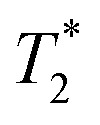
 sequences are more sensitive for tracking labelled cells, we compare 
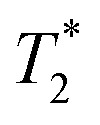
 relaxivity here (methods described in ESI†).^19^ For the stock solutions, the SPIONs prepared at *t* = 0 hours have a relaxivity of 452 mM^–1^ s^1^ and the SPIONs prepared at *t* = 5 hours have a value of 298 mM^–1^ s^–1^. The lower relaxivity for *t* = 5 h (maghemite) is expected given its lower saturation magnetisation. After 2 days in citrate buffer at pH 4.5, the 
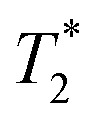
 effect of SPIONs prepared at *t* = 0 hours is completely lost, with its relaxivity decreased to 22 mM^–1^ s^–1^. The relaxivity for the SPIONs prepared at *t* = 5 hours, in contrast, only drops slightly to 270 mM^–1^ s^–1^, showing agreement with the dissolution data in Fig. 3a. MR images reflecting the change in contrast generation at 0.25 mM before and after exposure to pH 4.5 are shown as insets in Fig. 3b. Although relaxivity values are used here for comparative purposes of stability, the values may not reflect the actual dissolution in cells, for the reasons discussed above.

**Fig. 3:**
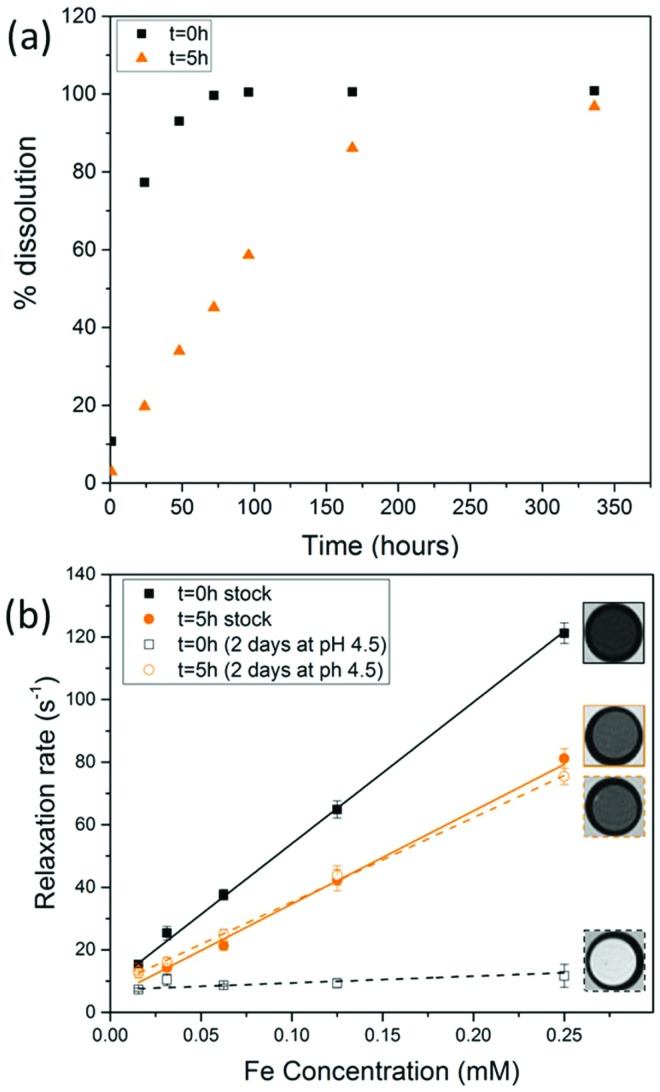
(a) Dissolution of SPIONs in citrate buffer system at pH 4.5 at 37 °C for 14 days (b) 
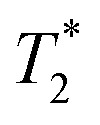
 solution relaxivity of SPIONs before and after exposure to citrate buffer for 2 days. MR images correspond to a concentration of 0.25 mM as imaged with a MGE sequence at TE = 15.5 ms.

To assess the suitability of these materials to label cells, we exposed a mouse mesenchymal/stromal stem cell line (ATCC CRL-12424) to a concentration of 10 μg mL^–1^ [Fe] of each SPION for a period of 24 h. This resulted in equivalent intracellular levels of ∼9.1 pg [Fe] per cell (Fig. 4a) for both samples, highlighting how their equivalent shell properties (size and zeta potential) lead to similar uptakes.

**Fig. 4:**
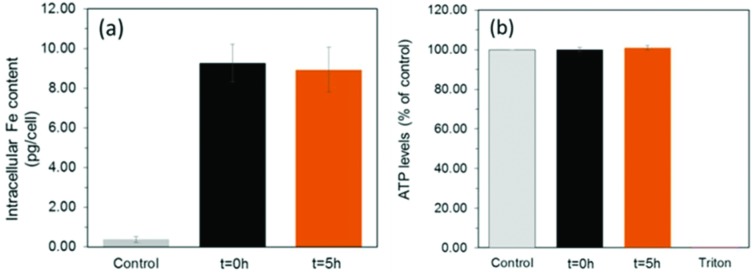
(a) Uptake (calculated probability *p* = 0.8 between the different particles) and (b) viability (*p* = 0.56 between the different materials) of MSCs labelled with 10 μg mL^–1^ SPIONs for 24 hours. Triton-X 100 (0.1%) was used as a negative control to induce cell death.

Importantly, this is an intracellular iron concentration that has previously been shown to yield strong MRI contrast *in vivo*.^27^ Viability measurements obtained by quantification of ATP (Fig. 4b, see ESI† for methods) revealed that neither of the two samples, irrespective of a core consisting predominantely of magnetite or maghemite, were toxic, with ATP levels equivalent to those found for controls (unlabelled cells).

We then sought to investigate the long-term stability of each SPION in lysosomes and, in particular, whether they agree with the data obtained with the citrate method shown as shown in Fig. 3b. For that purpose, we arrested the MSCs immediately after SPION labelling by exposure to Mitomycin-C (20 μg mL^–1^, 4 hours), and kept them in an humidified incubator for up to 14 days with regular medium changes.

Mitomycin-C is a DNA crosslinker that stops cell proliferation and thus prevents dilution of the SPIONs between daughter cells. We then compared how these MSCs generate MR contrast at three time points: directly after labelling, 2 days after labelling (*i.e.* equivalent to the conditions in Fig. 3b) and 2 weeks after labelling (Fig. 5).

**Fig. 5:**
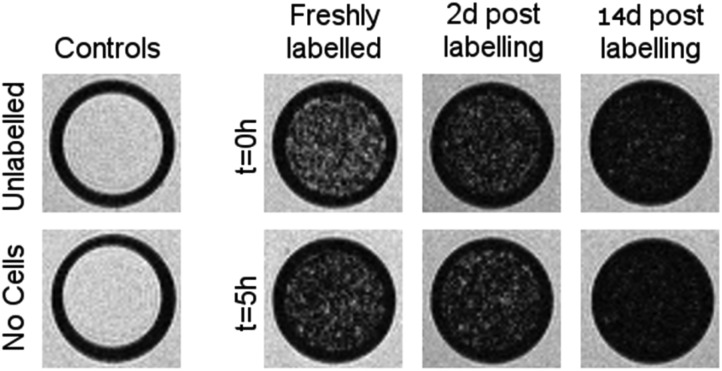
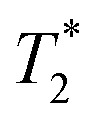
 weighted MR images of MSCs labelled with SPIONs at different time points. Controls consist of an equivalent number of unlabelled cells or agarose only.

Cells were harvested at each time point, fixed with formaldehyde, and then suspended in agarose at a concentration of 1.5 × 10^3^ cells per μL, which is equivalent to ∼0.25 mM of Fe when a uptake of 9.1 pg per cell is considered. Each of these samples was then imaged *via* MR with a 
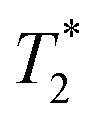
 sequence (described in ESI†), where any significant intracellular degradation of SPIONs should lead to a loss of negative contrast. Interestingly, at all 3 time points, the level of negative contrast is similar, irrespective of the SPION phase (*t* = 0 hours or *t* = 5 hours). Samples imaged 2 weeks post-labelling displayed a noticiable increase in signal loss, which is likely due to SPION transfer from dying cell debris to the remaining, viable, cells. It should be noted that a small fraction of cells undergo apoptosis during a two week culture period, and the uptake of SPION debris to viable cells has been previously shown in the literature.^29^ Strikingly, there is no indication of degradation for either of the SPIONs, regardless of whether the iron oxide core consists predominantely of magnetite or maghemite. This is in strong contrast to Fig. 3b, where the SPIONs that consist predominantely of magnetite (*t* = 0 hours) were nearly fully degraded and unable to generate contrast when placed in citrate buffer at pH 4.5 for 2 days. This highlights how the degradation kinetics can be extremely misleading under conditions that do not accurately reflect the environments found in cells. In fact, even the hypointense signal generated by cells 14 days post–labelling is similar for both sets of SPIONs, suggesting that irrespective of the predominant phase of the core, the SPIONs internalised in cells remain very stable for long periods of time. For example, we have previously shown that SPIONs used to label macrophages can generate MR signal *in vivo* for up to 1 month.^26^ The same observations were made when imaging the same samples using *T*_2_ weighted sequences (Fig. S7†).

## Conclusions

We have shown using UV-Vis-NIR, XANES and pXRD that it is possible to use co-precipitation to synthesise SPIONs that contain predominantly magnetite or pure phase maghemite and we have compared them for cell tracking purposes. Whilst a citrate buffer lysosomal mimic indicates that maghemite particles could be significantly more stable in the long term, in fact, when particles are internalised in MSCs their stability is largely the same as the predominantly magnetite containing SPIONs and the MR signal is retained for up to at least two weeks. Thus, it is not possible to establish any major differences between the MR performance of either phase in the time frame of most pre-clinical studies, particularly when imaging lasts less than 14 days. This could mean that when the production of these materials is considered, the extra oxidation step and synthesis time may not be required for cell tracking purposes. This study further indicates the need to characterise SPIONs and other nanomaterials before and after exposure to physiological conditions to better understand their performance *in vivo*.

## Conflicts of interest

There are no conflicts to declare.

## Supplementary Material

Supplementary information
