# Supplementary material for: SPIONs for cell labelling and tracking using MRI: magnetite or maghemite?
Source: Biomater Sci. 2017 Nov 23;6(1):101–6. doi: 10.1039/c7bm00515f (PMC5793703; doi:10.1039/c7bm00515f)
Supplement: Supplementary file 1 [file BM-006-C7BM00515F-s001.pdf]

Electronic Supplementary Material (ESI) for Biomaterials Science.
This journal is © The Royal Society of Chemistry 2017

SPIONs for cell labelling and tracking using MRI: magnetite or maghemite?

Michael Barrow[a]‡, Arthur Taylor[b]‡, Ana María Fuentes Caparrós[a], Jack Sharkey[b], Luke M. Daniels[a], Pranab Mandal[a], B. Kevin Park[c], Patricia Murray[b], Matthew J. Rosseinsky[a]*, Dave J. Adams[ad]*.

‡ Authors contributed equally

a. Department of Chemistry, University of Liverpool, Liverpool, UK

b. Centre for Preclinical Imaging, Institute of Translational Medicine, University of Liverpool, Liverpool, UK

c. MRC Centre for Drug Safety Science, Department of Clinical and Molecular Pharmacology, University of Liverpool, Liverpool, UK.

d. School of Chemistry, College of Science and Engineering, University of Glasgow, Glasgow, UK.

Emails: Dave.Adams@glasgow.ac.uk, rossein@liverpool.ac.uk .

Synthesis: Magnetite containing SPIONs (t=0 hours) were prepared using approximately 1:1 polymer to iron salt mass to mass ratio. 0.05 g DEAE-Dextran (Mw 40 000 Da), 0.03 g (1.1×10^{-4} mol) ferric chloride hexahydrate ($FeCl_3{\cdot}6H_2O$) and 0.015 g (7.5×10^{-5} mol) ferrous chloride tetrahydrate ($FeCl_2{\cdot}4H_2O$) were added in 25 mL water in a two necked flask fitted to a non-magnetic PTFE stirrer through an air-tight connection. The mixture was purged with nitrogen for 30 minutes on ice and 1 mL ammonium hydroxide (28-30%) was added dropwise into the solution over a period of 120 s whilst stirring at 200 (±5) rpm. The solution was then transferred from ice to an oil bath at 60°C and the temperature was increased to 80°C over a period of 15 minutes and kept at 80°C for 1h, whilst kept under nitrogen. To prepare pure maghemite particles (t=5 hours) following the procedure above the sample was opened to air, a reflux condenser was attached to the flask and the temperature was increased to 110°C for 5 hours.

Purification: Both solutions were cooled to room temperature and dialyzed using 100K membrane until the particle solution reached pH 7. The solution was concentrated using spin filters to around 1-2 mL in total and passed through dextran-based g100 Sephadex® size exclusion beads to separate any excess free polymer. Spin-filters were again employed to provide an extra washing step, the solutions were concentrated by centrifugation 3 times using 15 mL deionized water to remove any unreacted material and impurities and spun until a volume of 3-5 mL was left in the filter compartment. Water volumes were adjusted to yield stock solutions of around 1 mg ml^{-1} (iron basis) and passed through 0.22 µm polyethersulfone (PES) membrane to sterilise the solutions. Samples consisting predominantly of magnetite (t=0h) were stored under nitrogen to prevent oxidation during storage.

For samples synthesised with no polymer for XANES and FTIR, the particles were magnetically separated after synthesis, washed three times using distilled water and freeze dried.

Characterisation

Ultraviolet-visible-near infrared spectroscopy (UV-Vis-NIR)

Vis-NIR measurements in Figure 1 were carried out on a Hitachi High Technologies Corporation UV-Vis Spectrophotometer (U-3000).

Citrate data were collected on BioTek PowerWave HT Microplate Spectrophotometer.

XANES measurements

X-Ray absorption near edge measurements were made in transmission mode on beamline B18 at Diamond Light Source at the Fe K-edge. Pellets were prepared by grinding ~5 mg of each sample with 100 mg cellulose powder and pressing to a thickness of around 1 mm. Data were normalised using Athena software where linear pre-edge and polynomial post-edge backgrounds were subtracted.[1]

Citrate assay

The dissolution medium was a phosphate buffer saline solution containing 20 mM sodium citrate where the pH was adjusted to 4.5 using HCl. SPIONs were added to the solutions at a concentration of 5 µg/mL [Fe] and kept at 37°C. At each time point an aliquot was taken to measure free iron ions. A colorimetric reagent based on Ferrozine (65mM) and ascorbic acid (10 mM) was added to the buffer (20% by volume) and left do develop a blue colour for 30 minutes. Samples were transferred to a 96 well plate and the absorbance was measured at 590 nm with respect to calibration standards.

Powder X-Ray diffraction (pXRD)

Measurements were carried out on a Panalytical X'pert Pro multipurpose diffractometer with a Co Kα source (λ = 1.78 Å). Patterns were measured over 20–120° 2θ for 2 hours (step size 0.033°, time per step 295.3 s and scan speed 0.014 °/s). The mean core diameter (D_m) was calculated by using the fit profile tool (pseudo Voigt peak profile) on X'Pert Highscore plus software to obtain β, the full width half maximum (FWHM) of the 311 Bragg reflection and applying the Scherrer equation:

$$D_m = \frac{K.\lambda}{\beta.cos\theta_B}$$

where K = unknown shape factor (0.9), and θ_B = the Bragg angle. For the stacked Figure S1, the background was subtracted for clarity.

Dynamic light scattering (DLS) and zeta potential (ZP)

Measurements were carried out on a Malvern Zetasizer Nano ZSP instrument (Sigma, Dorset, UK). ZP measurements were carried out in 0.01 M NaCl solution.

Thermogravimetric analysis (TGA)

Measurements were carried out on a TA Instruments SDT Q600 TGA machine using a constant air flow of 50 mL min^{-1}. Samples were heated up to 120 °C at a heating rate of 20 °C min^{-1} and kept at 120 °C for 20 minutes to remove any water, then ramped up to 1000 °C at a heating rate of 10 °C min^{-1}.

Magnetic measurements

Magnetic isotherm data were collected at 300 K in DC scan mode (scan length 30 mm, scan time 4 seconds, 2 scans per measurement) using a MPMS3 SQUID (superconducting quantum interface device) magnetometer (Quantum Design). Freeze-dried samples (0.5–1.5 mg) were packed in a size 4 gelatin capsule and fixed in a plastic drinking straw.

MR Methods

All MR data was acquired with a Bruker Avance III spectrometer interfaced to a 9.4T magnet system (Bruker Biospec 90/20 USR) using a 40 mm transmit/receive volume coil. Relaxivity was calculated from the relaxation times obtained with a multi gradient echo sequence whereas imaging of cells was carried out with a FLASH sequence. In all cases, particles or fixed cells were suspended in 1% (w/w) low gelling temperature agarose and allowed to set in tubes with a volume of 80 µL, which were subsequently mounted in a larger holder containing agarose. For cell measurements cells were suspended at 1.5×10^3 cells/µL, which is equivalent to 0.25 mM of Fe when an uptake of 9.1 pg [Fe]/cell is considered. Imaging parameters are listed below in Table S1

Table S1 MR Acquisition parameters

Sequence	MGE T_2^* Map	FLASH T_2^*	RARE T_2
Purpose	T_2^* relaxation	T_2^* Imaging	T_2 Imaging
Echo Time (ms)	4.5, 10, …, 43 (8 echoes)	6.5	37
Repetition Time (ms)	800	200	2200
Flip Angle	50°	20°	90° / 180°
Averages	2	100	100
Matrix Size (pixels)	256x256	400x400	400x400
Field of View (mm)	30x30	30x30	30x30
Slice Thickness (µm)	1000	160	160
Acquisition Time	5 min.	2h 13 min.	3h 3 min.

Ferrozine methods

Cell culture, SPION labelling and ferrozine quantification was carried out as previously described.[2]

Cell Viability:

Cell viability was measured using the CellTiter-Glo® reagent (Promega). Briefly, 10^4 murine mesenchymal/stromal cells (MSC D1 line, ATCC CRL-12424) were seeded overnight in each well of a 96-well plate. Those were then incubated for 24h with each of the SPIONs suspended in culture medium or 0.1% Triton-X 100 as a negative control. After this period the medium containing SPIONs was replaced with 50 µL fresh medium and 25 µL CellTiter-Glo® reagent. This was followed by orbital mixing (2 minutes), incubation (10 minutes) and luminescence measurement using a Fluostar Omega (BMG Labtech) plate reader.

Freeze dryer

Samples for TGA and SQUID measurements were freeze dried using a Labconco freezone 4.5 freeze-dryer with a condenser temperature of –50 °C and a shelf temperature of 20 °C.

Materials

Diethylaminoethyl (DEAE)-dextran (Mw 40,000 Da), Iron (III) chloride hexahydrate $FeCl_3$.6(H2O) (ACS reagent, 97%), Iron (II) chloride tetrahydrate $FeCl_2.4(H_2O)$ (reagent plus®, 98%), ammonium hydroxide solution (28–30 %), Corning® Spin-X® (100 k), sodium azide, Sephadex® G-100 beads, 3-(2-Pyridal)-5,6-di(2-furyl)-1,2,4-triazine-5',5"-disulfonic acid disodium salt (Ferrozine reagent), sodium citrate, PBS tablets, L-ascorbic acid, low gelling temperature agarose (2-Hydroxyethyl agarose), Iron (II,III) oxide and Iron (II) oxide were all purchased from Sigma Aldrich (Dorset, UK). Spectra/Pore Biotech cellulose ester (CE) dialysis membranes (MWCO: 100,000 Da) and Millex GP syringe filters with a polyethersulfone (PES) 0.22 µm membranes were purchased from Fisher Scientific (Loughborough, UK). Deionised water from a Milli-Q system (Millipore Limited, Hertfordshire, UK) (resistivity 15 MΩcm at 25 °C) was used for all experiments.

Supporting Figures

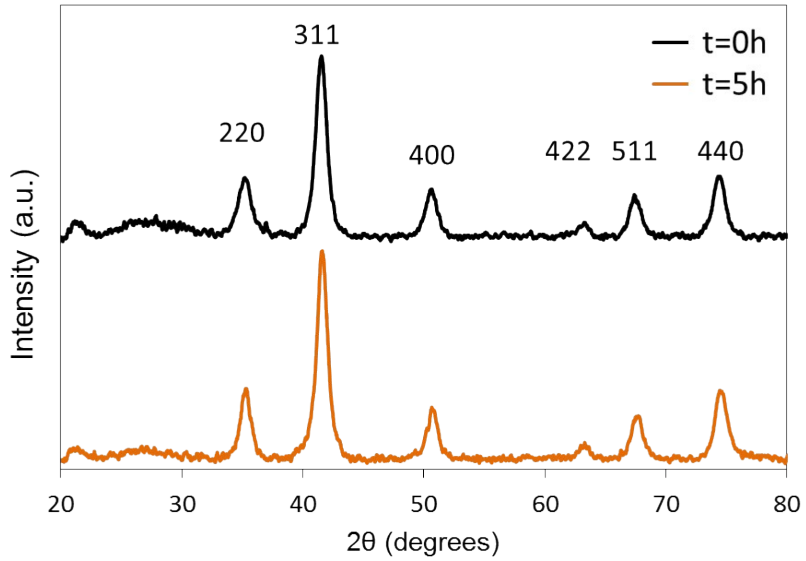

Figure S1 pXRD patterns of SPIONs measured using CoK_α radiation (after background subtraction).

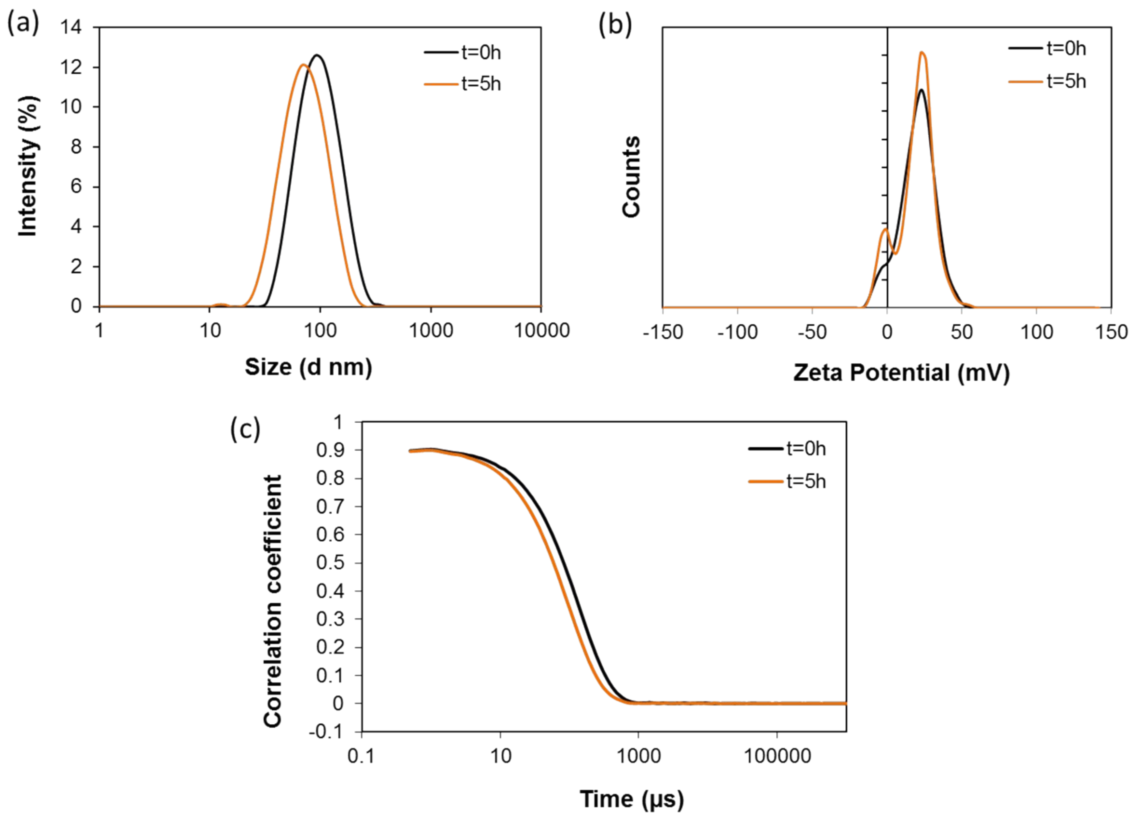

Figure S2 (a) Particle size distribution of SPIONs as measured by DLS and (b) apparent zeta potential measurements of both obtained in 0.01M NaCl solution at room temperature and (c) correlation functions.

Table S2 Particle size measurements by DLS with polydispersity index (PDI), zeta potential values and standard deviation.

**Sample**	**Hydrodynamic Z-Avg in 0.01 M NaCl (nm)**	**PDI 0.01M in NaCl**	**Zeta Potential in 0.01M NaCl (mV)**	**Standard deviation (mV)**
t=0 hours	90.2	0.157	19.9	± 11.8
t=5hours	64.4	0.169	19.0	± 12.1

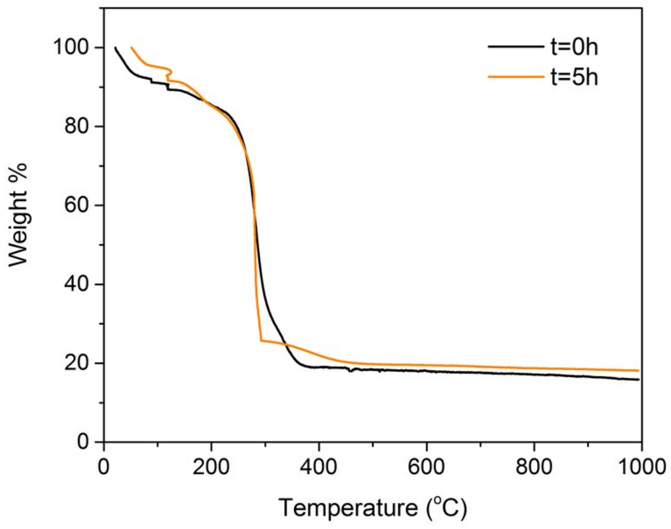

Figure S3 Thermogravimetric analysis (TGA) weight loss curves for t=0h and t=5h SPIONs measured in under an air flow.

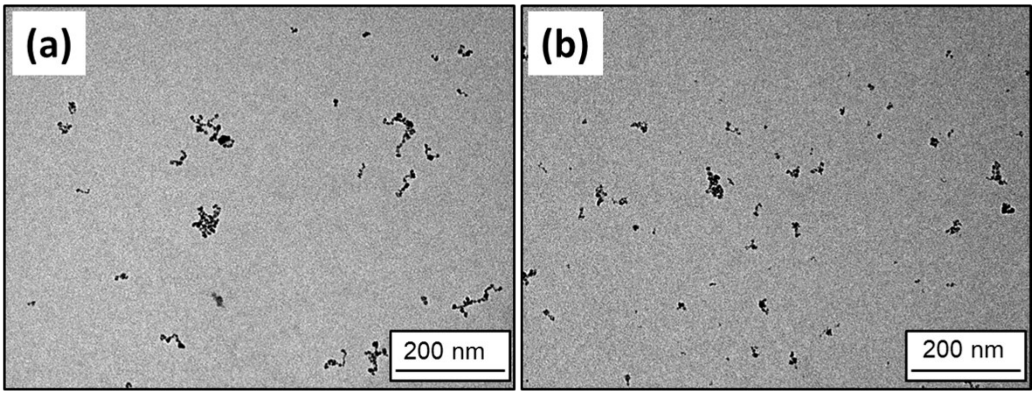

Figure S4 TEM images of (a) t=0 hours and (b) t=5 hours polymer coated SPION samples

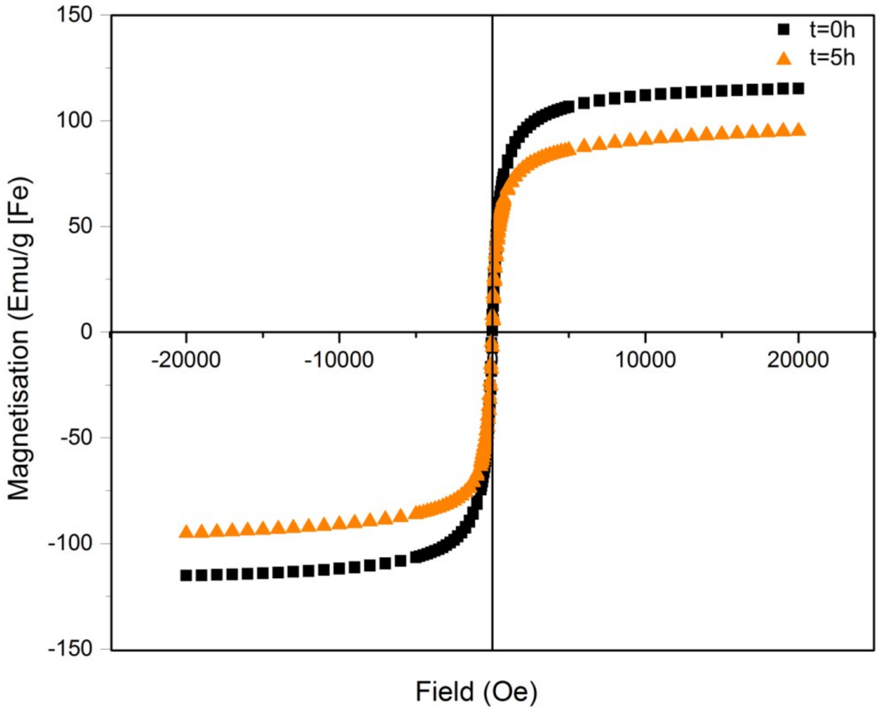

Figure S5 Magnetisation curves for as produced SPIONs measured at 300K.

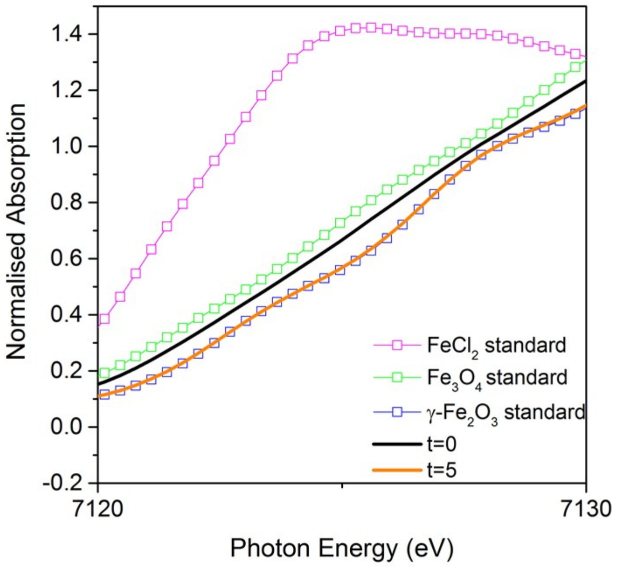

Figure S6 Magnified comparisons of Fe K-edge energies.

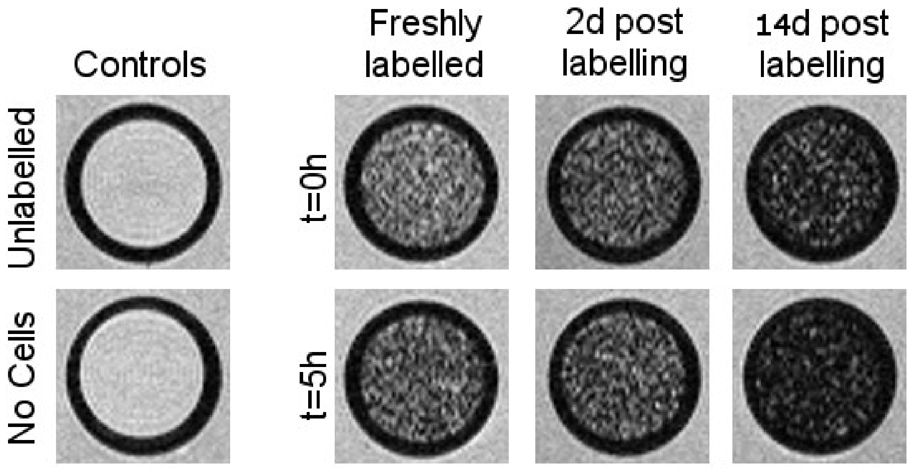

Figure S7 T_2 weighted MR images of MSCs labelled with SPIONs at different time points.

References

1. B. Ravel and M. Newville, *Journal of Synchrotron Radiation*, 2005, **12**, 537-541.
2. M. Barrow, A. Taylor, J. García Carrión, P. Mandal, B. K. Park, H. Poptani, P. Murray, M. J. Rosseinsky and D. J. Adams, *Contrast Media & Molecular Imaging*, 2016, **11**, 362-370.
